# Morphospace, Responsibility and The Ethics of Form: A Model for Sustainable and Ethical Design

**DOI:** 10.1007/s11948-026-00582-3

**Published:** 2026-02-09

**Authors:** Marco Tamborini

**Affiliations:** TU Darmstadt, Institut für Philosophie, Residenzschloss 1, Darmstadt, 64283 Germany

**Keywords:** Ethics, Robotics, Architectural design, Evolution, Philosophy of science and ethics in practice, Bioengineering

## Abstract

This paper proposes the integration of an ethical dimension into the concept and design of morphospace. This is traditionally used in paleontology, architecture, and bioengineering to visualize and generate theoretically possible forms given certain parameters. Drawing from *Value-Sensitive Design* (VSD) and *Responsible Research and Innovation* (RRI), I introduce a structured methodology to quantify and operationalize ethical considerations in *morphospace*. While current applications of morphospace focus on geometric and technological parameters, in this paper I propose a methodological extension that incorporates criteria of environmental sustainability and social impact. By intersecting value-sensitive design and bioinspired design, the paper develops therefore an approach that allows for the evaluation of engineered forms not only in terms of technical feasibility but also ethical responsibility. Moreover, the paper also highlights the need for interdisciplinary governance to constantly update ethical standards in bioengineering and architectural design. Finally, I discuss key challenges, including interdisciplinary standardization, computational complexity, and evolving ethical standards for implementing the ethical dimension in morphospace’s design. By transforming morphospace from a purely feasibility-driven model to one that incorporates ethical responsibility, this work aims therefore to bridge the gap between technological possibility and value-sensitive-practice in design and engineering.

One of the key challenges in contemporary design is the creation of engineered forms that are not only highly efficient for their designed purpose but also aligned with ethical and ecological principles. In several engineered and bio-inspired disciplines that have been developed in recent years, such as biomimetics and biomimicry (Dicks, 2023; Gerola et al., 2023; Tamborini, 2022a), this need is embedded in their call for sustainable and bio-inspired design (Antony et al., 2014; Barthlott et al., 2016; Bensaude-Vincent, 2019; Blok, 2023; Gilbert, 2021; Giordano et al., 2023; Tamborini, 2024c, 2023). For instance, the ethical principle of biomimicry is based on seeing “nature as model” and “nature as measure” (Benyus, 2002). Biomimicry has indeed pushed a profound ethical framework. As philosopher Henry Dicks has extensively elaborated, the ethics of biomimicry extends beyond mere technological innovation: it calls for a shift in humanity’s relationship with nature. At its core, biomimetic ethics emphasizes sustainability, interconnectedness, and a commitment to preserving a habitable Earth for all living beings (Dicks, 2023, 2016; Tamborini, 2025b; Blok, 2017).

Alongside the metaphysical principle of taking nature as a “measure,” various responsible design approaches have explored how to integrate ethics and values into design in a sustainable manner. Among them, Value-Sensitive Design seeks to embed human principles and values within the design process to come up with value-sensitive design best practices (Umbrello & de Poel, 2021; Umbrello, 2020; Friedman, 1996; Friedman et al., 2002; Friedman & Hendry, 2019).

This paper aims to expand the discussion on Value-Sensitive Design and bioinspired design. In the following pages, I propose a methodology to merge these approaches by incorporating ecological and biological principles. By situating my analyses within the broader philosophy and ethics of science and technology in practice–that is, a philosophical and ethical reflection on what scientists actually do –I propose to introduce an ethical parameter in the design of the morphospace, a practice widely used in design. This addition would help visualize possible engineered forms that also align with the ethical values defined by the design community.

In recent decades, the concept of morphospace has been applied across various scientific and technological fields^1^. Originally developed in paleontology during the 1960s to describe and examine the diversity of organic forms that can (or could, in purely theoretical terms) exist (Raup & Michelson, 1965; Raup, 1962; Sepkoski, 2012), morphospace has since been adopted in disciplines such as digital architecture and bioengineering, among others. Through a process of knowledge circulation and hybridization, architects and engineers have adapted and redefined the paleontological morphospace to guide the production of engineered forms (Tamborini, 2022c). This theoretical tool allows the representation of all possible forms of a given system in a multidimensional space. Based on that, scientists can identify which forms exist in nature, which could exist but have not evolved, and which are structurally or functionally impossible to manufacture.

In the fields of robotic and architectural fabrication, morphospace^2^ has been employed to explore construction possibilities and optimize design and production processes. For example, in 2013, German architect Achim Menges showed how morphospace could be transferred from biology to computational architecture, creating a model that considers both theoretically realizable geometries and the limitations imposed by robotic fabrication technologies (Menges, 2013). This approach enables a precise identification of which architectural forms can be effectively constructed through robotic fabrication.

Similarly, in bioengineering (particularly in the development of organoids and artificial organs) morphospace has been used to identify biologically feasible configurations. In a study published in *Integrative Biology* in 2016, scientists Aina Ollé-Vila, Salva Duran-Nebreda, Núria Conde-Pueyo, Raúl Montañez, and Ricard Solé investigated how morphospace could be applied to predict new cellular and tissue structures that do not exist in nature but could be synthesized through tissue engineering and bioprinting technologies (Ollé-Vila et al., 2016).

Despite the merit of these approaches–among them the transfer and implementation of purely biological, paleontological and morphological procedures (i.e. morphospace) into bioinspired design–, a key challenge remains: the lack of methodological discussion and, ultimately, expansion to include ethical parameters in the visualization of possible producible forms through morphospace.

Indeed, today’s morphospace*s* consider only geometric, structural, and technological parameters. It does not incorporate ethical or social criteria. This raises the fundamental questions at the heart of this paper: should we design every shape that is technically possible, or should we also set ethical constraints? If we opt for responsible and value-driven design, how can we responsibly extend the design of morphospace to take into account ethical and social values and issues?

This paper seeks to address these issues by introducing an ethical dimension into morphospace, with the goal of assessing the social and environmental impact of designed forms. The central thesis is that architectural and bioengineering design cannot be limited to purely technical considerations; rather, it must also incorporate reflections on sustainability, social equity, and the long-term implications of technological innovation.

To develop this, I proceed as follows: first, I briefly present the use of morphospace in architecture and bioengineering; second, I show which dimension is missing from such a use of morphospace; third, I develop the ethical parameters applicable in morphospace; fourth, I attempt a preliminary operationalization^3^ my proposal for integrating ethical evaluation into morphospace. In the outlook, I reflect on the broader payoff and challenges of my proposal.

## Morphospace in Architectural Design and Bioengineering

The concept of morphospace was coined by the American paleontologist David Raup in the 1960s (Raup & Michelson, 1965; Raup, 1962; Sepkoski, 2012; Tamborini, 2022b). Using an analog computer (PACE TR-10) and an oscilloscope, he created and visualized a virtual space—i.e., the morphospace—where, based on certain physical parameters, he could generate all theoretically possible forms of coiled shells. These parameters included: first, the shape of the generating curve; second, the rate of expansion of this curve; third, the amount of overlap between successive whorls, and fourth, the position of the whorl relative to the axis (Raup, 1967).

Building on this geometric model of form production and using computational tools, Raup developed morphospace as a means to “visualize the total spectrum of geometrically possible shell forms. This spectrum takes the form of a four-dimensional space, with one dimension for each of the basic parameters. It is assumed that each parameter is independent of the others and that all combinations of the four are possible” (Raup, 1967, p. 1181).

**Fig. 1:**
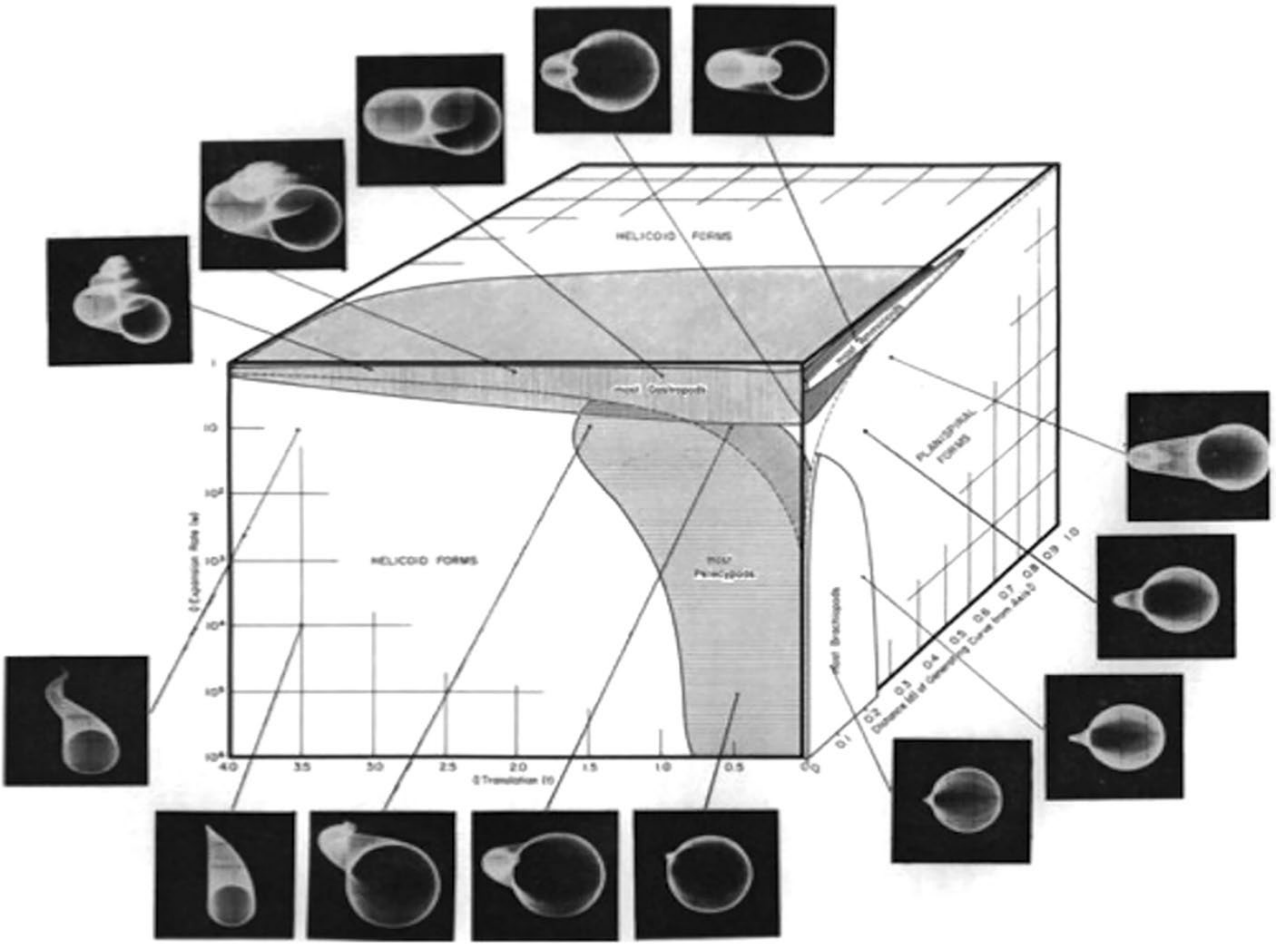
Raups’ morphospace visualizes all possible forms that could be theoretically produced following the previously identified parameters (Raup, 1967, p. 1184). Reprinted with permission from SEPM

This marked a turning point in morphological research, as it introduced the possibility of visualizing and controlling morphogenesis, i.e., the principles responsible for form production. For instance, Raup developed a computer program to simulate the plate configuration of the *echinoid ambulacrum*^4^, the fossilized remains of sea urchins. Morphospace, therefore, serves as a digital visualization of all the forms that could theoretically be produced based on the previously defined parameters (see Fig. 1).

An important distinction out of Raup’s analyses must be emphasized: a theoretical morphospace is constructed a priori, while an empirical morphospace is derived from real-world data. This distinction is crucial, as philosopher Adrian Currie puts it: “a *theoretical* morphospace is constructed a priori, while an empirical morphospace is constructed from data. This difference matters: the modal profile of a theoretical morphospace is broader in scope, while an empirical morphospace is grounded in the real world. Moreover, a theoretical morphospace, as it relies on the intuitions of the on the modeler, is open to worries of mind dependence, while empirical morphospaces are worryingly sensitive to the selected data set” (Currie, 2012, pp. 586–587).

Thus, morphospace functions as a method for visualizing and generating forms based on specific parameters responsible for their production. However, it does not generate forms in a complete or deterministic manner. As Raup himself acknowledges, and it is essential to stress this point:

It should be emphasized that the four parameters do not completely describe the morphology of the coiled shell. The complete morphological picture would include many features such as surface ornamentation, growth rings, internal structure, shell thickness, and so on. The model is concerned only with general form […] Furthermore, the model does not describe ontogenetic change in coiling, though this can sometimes be added as an elaboration of the basic equations (Raup, 1967, p. 1181).

Thus, through morphospace design, scientists can produce and visualize forms in an inherently incomplete manner, leaving open the possibility of further morphological and evolutionary development. As in the case of Raup’s coiled shells morphospace, features such as surface ornamentation, internal structures and growth dynamics are not inherently encoded in the model, allowing for continuous refinements and extensions in its application.

### Morphospace in Architectural Design

In the field of digital fabrication and robotics applied to architecture, morphospace is used to map all possible geometries that can be constructed using numerically controlled machines and industrial robots^5^. As analyzed in previous articles, British architect Philip Steadman, in a series of papers written with Linda J. Mitchell, implemented Raup’s morphospace to map and classify possible construction plans (Steadman & Mitchell, 2010; On this see; Tamborini, 2022c). Successively, in 2013, architect Achim Menges introduced morphospace into architectural design, expanding its application beyond its biological origins.

Menges identifies two main levels within morphospace: the theoretical morphospace, defined as the set of all geometrically possible forms in theory, regardless of their manufacturability; the machinic morphospace, identified as the subset of morphospace representing the forms that can actually be realized using a specific set of tools and robotic fabrication technologies (Menges, 2013).

**Fig. 2:**
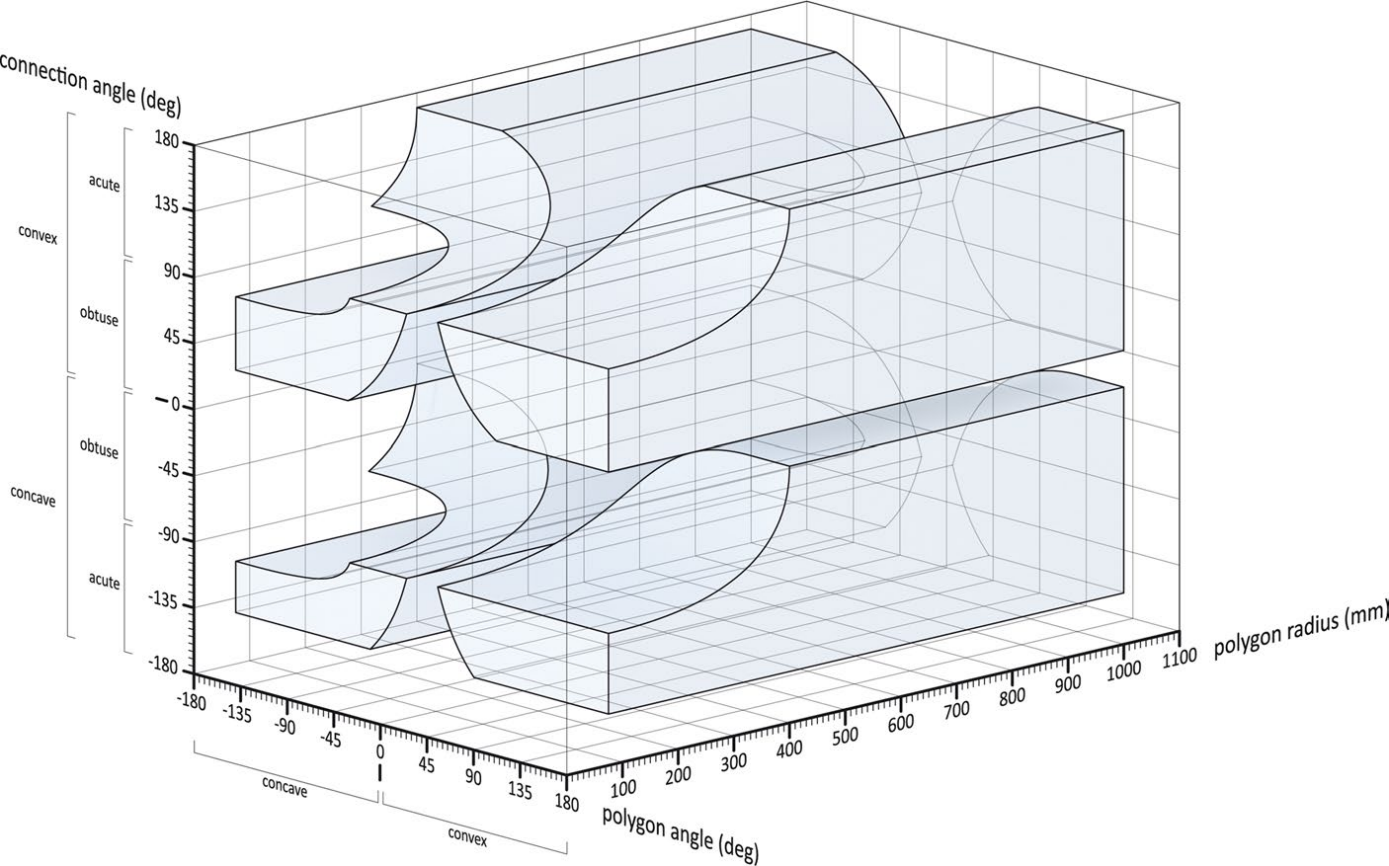
Menges’ machinic morphospace (Menges, 2013, p. 42). Reprinted with permission of the author

A practical example of morphospace application in robotic fabrication is the study of comb joints for wooden panels. In architecture, comb joints are used to efficiently connect structural elements without the need for nails or glue (see Fig. 2). Menges showed that by leveraging morphospace, it is possible to optimize the geometry of these joints, adapting them to the constraints of robotic fabrication and improving their structural performance (Menges, 2013).

The adoption of this method has led to the development of lighter, stronger, and more efficient constructions, with reduced material waste. However, one significant limitation of the current approach is that it does not directly consider the environmental impact of production within the morphospace design framework.

### Morphospace in Bioengineering and Organoids

In the field of bioengineering, morphospace is used to explore theoretically possible biological forms and compare them with those existing in nature. For example, a study published in *Integrative Biology* in 2016 (Ollé-Vila et al., 2016), scientists Aina Ollé-Vila, Salva Duran-Nebreda, Núria Conde-Pueyo, Raúl Montañez, and Ricard Solé investigate in detail how this concept can be applied to the design of organoids and artificial organs. They design a morphospace based on three key parameters: First, developmental complexity (“The degree of complexity introduced by developmental mechanisms required to build the structure” (Ollé-Vila et al., 2016)); second, physical state (“The level of cellular interaction and physical coherence of the structure under consideration” (Ollé-Vila et al., 2016)) this second parameter “introduces a well-known concept from physics: states of matter, here reduced to two of them (solid and liquid)”; third, Cognitive complexity (“The amount of information that a system is able to learn, store, and process” (Ollé-Vila et al., 2016))

**Fig. 3:**
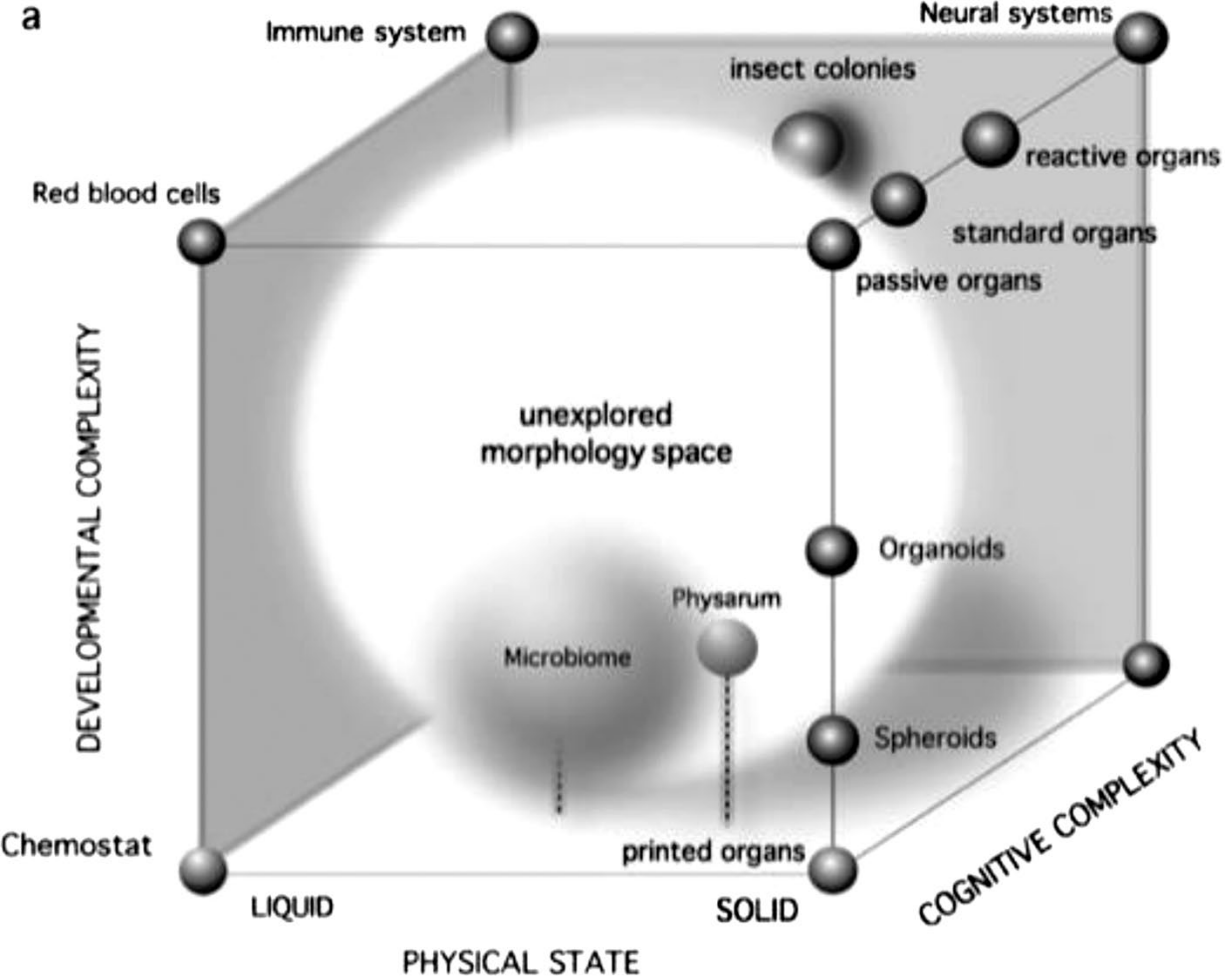
Aina Ollé-Vila’s and colleagues’ schematic depiction of tissue distribution within the proposed three-dimensional organ morphospace. Reprinted with authors’ permission (Ollé-Vila et al., 2016)

The authors propose that “organs and organoids should also be considered as cybernetic systems incorporating the three elements of any homeostatic structure: sensors, comparators, and actuators” (Ollé-Vila et al., 2016). Under this framework, “for a lower cognition system, these self-sustaining cell cultures exhibit no particular class of behavioral response beyond cell maintenance. At the other end of the spectrum, the immune system or the brain is able to process information from multiple sources, using very rich loops involving multiple scales” (see Fig. 3) (Ollé-Vila et al., 2016).

Beyond presenting a unified framework for integrating synthetic biology and tissue engineering to explore the uncharted regions of morphospace, this study clearly shows how to design a morphospace as a “simplified universe of possibilities” and how to address both the populated and unpopulated “areas inhabited by known organs and some designed organoids” (Ollé-Vila et al., 2016).

Hence, from these three cases, morphospace design is based on three interconnected phases: first, generation: the computational or algorithmic process by which parameter combinations are mapped to candidate forms (analytical formulae, as in Raup’s shell model); second, visualization: translating the high-dimensional parameter/form information into human-interpretable displays; and third, selection: the phase where candidate forms are chosen for evolutionary studies or, in architectural design, for prototyping or production.

## What is Missing?

As the three emblematic cases exposed in the previous section show, the use and design of morphospace is based largely (or rather exclusively) on geometric and technological criteria. However, this approach overlooks three fundamental issues at the core of contemporary philosophical and ethical debates on technology:

1. Epistemological issue: Morphospace assumes that every possible form is neutral, yet reality shows that designed forms have cultural, social, and ecological impacts. In philosophical terms, every technology game is always embedded within a broader form of life (Coeckelbergh, 2018; Tamborini, 2024a).
2. Ethical issue: If we can visualize a good-adapted form in morphospace, does that mean we should design and manufacture it? Or should we first reflect on the intrinsic responsibilities involved in selecting and designing a form within morphospace? Architectural and bioengineering innovations can lead to social inequalities or environmental harm. These implications should be evaluated *ex ante*, not only when a technological product is released but also during its initial prototyping phase^6^.
3. Meta-Ethical issue: What criteria should guide design choices? The current system tends to marginalize fundamental questions regarding the moral responsibility of design.

For instance, in the architectural design, robotic fabrication could reduce material consumption but may also fundamentally transform the role of the architect. In bioengineering, the creation of organoids could revolutionize medicine but also raise concerns related to experimentation and the very definition of life.

To address these critical issues, I propose the integration of an additional ethical dimension into morphospace. This dimension would enable the evaluation of forms not only based on their technical feasibility but also on their environmental sustainability and social impact. In doing so, this proposal aims to expand the practices and insights of Value-Sensitive Design by demonstrating how design and ethical values can be meaningfully integrated^7^.

## The Ethical Parameter in the Morphospace

As previously discussed, the concept of morphospace has been developed as a tool to explore all theoretically possible forms within a given context (see above the notion of theoretical morphospace). However, neither in robotic fabrication nor in organoid bioengineering-two fields where morphospace plays a crucial role—has the ethical dimension been considered during the design and exploration phase. This omission is problematic, as it implies that every possible form is ethically neutral, reducing ethical evaluation to a mere ex-post consideration. As German philosopher Ernst Cassirer^8^ has already noted in the 1930s: „In this construction of the realms of will and the basic convictions upon which all moral community rests, technology can only ever be a servant, never a leader. It cannot by itself determine the goal, although it can and should collaborate in carrying it out. It best understands its own meaning and its own narrative when it is content in the fact that it can never be an end itself. Rather, it has to fit itself into another ‘realm of purpose’“(Cassirer, 2012, p. 49).

The construction of digital architectures and synthetic organoids-two examples discussed here—has direct consequences for society and the environment, thus necessitating an “*Ethisierung der Technik*” (“‘ethicization’ of technology“) (Cassirer, 2012, p. 49). Expanding on Value-Sensitive Design, this section proposes incorporating a debate on and selection of ethical values and principles within the design process itself. In other words, I advocate for the integration of an ethical dimension into *morphospace*, enabling the evaluation of forms not only based on technical, economic, and engineering feasibility but also on their environmental and social impact.

### Ethical Dimension and Criteria

The proposed ethical dimension is structured around two core criteria. These are not fixed principles but rather adaptable parameters that can evolve through rational deliberation and justification. Just as in any theoretical morphospace, these ethical parameters rely on the modeler’s intuitions and are open to concerns of mind-dependence (see above). However, this mind-dependence is not a shortcoming but a strength, as it fosters dialogue among stakeholders regarding which ethical aspects should be prioritized (as discussed in the conclusion of this paper). Moreover, by supporting non-fixed criteria, the ethical morphospace establishes a flexible methodology adaptable to diverse design perspectives. This aligns with an ethical pluralism rooted in the multiplicity of possible “technological games” and “forms of life” (Coeckelbergh, 2018, 2020; Tamborini, 2024a), advocating thus for a multi-criteria framework in which different ethical values are weighed based on the application context (Friedman et al., 2013; Davis & Nathan, 2015). For example: in bioengineering, distributive justice may take precedence over technological innovation in defining ethical parameters; whereas in architecture, sustainability might outweigh economic efficiency as the dominant criterion.

### First Criterion: Environmental Sustainability

The first ethical value to be embedded as a morphospace parameter is *environmental sustainability*: assessing the ecological impact of production and material usage. This approach explicitly connects two bioinspired design methodologies (architectural design and synthetic biology/tissue engineering) with the broader ethics of biomimicry. As Henry Dicks articulates, the fundamental principle of biomimicry is: “Being like or emulating Gaia, and that in turn gives rise to the following ethical maxim: *an action is good if it tends to participate in the provision and maintenance of a habitable Earth for its living inhabitants; it is bad if it tends otherwise*” (Dicks, 2023, p. 196).

This principle *translates* into a sustainability parameter within morphospace. For example, robotic fabrication must account for energy consumption, and organoid cultivation must consider potential pollution from biotechnology waste.

### Second Criterion: Social Impact

The second ethical parameter involves assessing the *accessibility* of new technologies to different socio-economic groups, their effects on labor markets (e.g., the replacement of human labor by robots or reduced demand for human organ transplants), and potential inequalities in access to organoids and synthetic organs. Once these parameters are selected, they must be *translated* into computational models. In architecture, for instance, sustainability could be quantified through energy consumption metrics or the use of recyclable materials. In bioengineering, equity in technology access could be for example incorporated into modeling criteria.

By integrating this new ethical dimension, morphospace enables not only an evaluation of which forms can be realized but also which forms should be built based on principles of justice and equity. In this sense, the ethical parameter functions as an *evolutionary constraint* (Gould, 1980; Brigandt, 2015; Amundson, 1994; Tamborini, 2022b): they limit some possible design pathways while opening new opportunities for evolvability.

### Ethical Constraints as Evolutionary Parameters

Like other morphospace parameters, the ethical parameter proposed here functions as a *possible constraint*. This happens in two ways:

1. A constraint *restricts* certain morphological variations, illustrating the impossibility of assuming a form is optimal based solely on natural selection.
2. A constraint serves as a crucial *driver* of evolvability in design.

In evolutionary biology, evolvability is defined as “the ability of organisms to generate heritable and viable phenotypic variation, which forms the mechanistic basis of morphological change” (Brigandt, 2015, p. 309). Within the ethical morphospace, evolvability refers to a form’s ability to mutate and adapt over time. As proponents of *Responsible Research and Innovation* argue, this necessitates “taking care of the future through collective stewardship of science and innovation in the present” (Stilgoe et al., 2020, p. 1570).

In addition, by incorporating ethical considerations as formal design parameters, morphospace operationalizes the three value functions outlined in Value-Sensitive Design (Umbrello & de Poel, 2021; Umbrello, 2023, 2024): the values to be promoted are materialized by constraints understood as drivers of evolvability; the values to be respected and contextualized are assumed by constraints as limits that shape the design space.

Finally, incorporating ethical constraints into *morphospace* aligns with Brigandt’s key observation: “Constraints can result in spandrels, i.e., the adaptive evolution of one trait entailing another trait as a developmental by-product” (Brigandt, 2015, p. 312). By producing spandrels (Gould & Lewontin, 1979), ethical morphospace fosters co-adaptation and co-evolution between designed forms and their users. In other words, it preserves the possibility for new interactions and emergent ethical considerations even after a design has been realized. This ensures that *ethical reflection remains an ongoing process rather than a static precondition in technological development*.

### Integration of the Ethical Dimension into Morphospace^9^

By incorporating an ethical dimension into the morphospace, it becomes a space in which forms with a high negative impact on the environment and society are placed in a critical zone, while ethically sustainable forms occupy a more favorable area. As in all morphospaces, void zones emerge. These are spaces with greater potential for ethical-technological innovation.

In architectural design and robotic fabrication, the ethical morphospace could help address practical challenges arising from automation. For instance, it could guide material and geometric selections based on sustainability criteria, ensuring a balance between automation and job retention in the construction sector. The same applies to bioengineering: an ethical morphospace could be used to identify organoids with minimal risk of uncontrolled proliferation, ensure equitable access to artificial organs, and address other ethical concerns.

Thus, the integration of the ethical dimension allows for the selection of forms that balance technical feasibility with moral responsibility, in effect fostering a more conscientious and responsible approach to design and innovation. How is it possible to operationalize the ethical constraints (as defined above) in the morphospace? In the next section I will provide tentative answer, thus calling for interdisciplinary collaboration.

## Operationalizing Ethical Constraints in Morphospace

To move beyond abstract ethical considerations, in effect embracing the ideas of Value-Sensitive Design, in this section I seek to *tentatively propose* how to operationalize the ethical constraints to integrate ethical evaluation into morphospace. These constraints should be defined based on three key methodological steps:

### Step 1: Selection of Ethical Parameters

Before integrating ethics into morphospace, we must define which ethical dimensions, i.e., constrains, are relevant. Drawing from Value-Sensitive Design and Responsible Research and Innovation, the most relevant parameters could include:

- Environmental sustainability: Carbon footprint, resource consumption, recyclability of materials, etc.
- Social impact: Accessibility, labor market implications, equity in technology distribution, etc.
- Biosafety and security: Potential risks of self-replicating organoids or unintended consequences in synthetic biology, etc.

These parameters are not universally valid, but must be context-dependent (or, in philosophical terms, they should depend on the technology game that scientists play (Coeckelbergh, 2018)). For example, in robotic fabrication, energy efficiency and material sustainability may take precedence; while in bioengineering, safety and accessibility of artificial organs could be the dominant ethical concerns. In this way, the constraints’ weight vary according to the kind of context, task, and form scientists are dealing with (exactly as constraints’ weight vary in evolution. See (Tamborini, 2022b)).

### Step 2: Quantification and Measurement of Ethical Criteria

The second step is about establishing quantitative metrics evaluating the ethical parameters chose within *morphospace*. To accomplish this, interdisciplinary work between philosophers, engineers and scientists is fundamental. As in the previous section, some tentative quantifiable metrics may include (see Table 1):

**Table 1:** Tentative quantifiable metrics that may be included into the morphospace

Ethical Parameter	Quantifiable Metric	Example
Environmental Impact	Carbon footprint (kg CO₂), energy consumption (kWh)	Evaluating robotic fabrication processes in architecture
Social Accessibility	Affordability index, population reach (%)	Assessing whether synthetic organs are accessible to different socio-economic groups
Biosafety	Risk assessment score (low, medium, high)	Identifying risks in self-replicating biological structures

These parameters can be integrated into a multi-dimensional scoring system, where each form generated in *morphospace* is assigned an Ethical Feasibility Score based on its conformity to predefined ethical thresholds.

### Step 3: Computational Implementation in Morphospace Models

Once ethical constraints are quantified, they must be *translated* into the computational structure of morphospace. This ensures that morphospace does not merely consider what can be designed but also what should be designed.

Ethical considerations can intervene at any of these stages, and the implications vary depending on where they are applied. When applied during generation, they act as hard *ex ante* filters: the parameter space is constrained so that every candidate already meets baseline thresholds, such as carbon limits or biosafety scores. If introduced during visualization^10^,, generation remains open, but each candidate is annotated with ethical metrics—such as carbon footprint, affordability, or recyclability—and these data are rendered through heatmaps or overlays. When ethical considerations are implemented at the selection stage, they become part of the decision process itself: they may be aggregated into an Ethical Feasibility Score or used in Pareto-front analyses, enabling nuanced balancing of competing values but also requiring consensus on metrics and weights.

## Outlook

In this work, I have argued that the absence of ethical criteria in current use and design of morphospace represents both an epistemological and ethical gap that must be addressed. I have therefore proposed the introduction of an ethical dimension into theoretical morphospace, marking a step toward making architectural and bioengineering design more in line with Value-Sensitive Design.

As argued in previous sections, traditional morphospace considers only geometric and technical constraints, while overseeing ethical impact. The addition of an ethical dimension would allow for the evaluation of forms not only based on their feasibility but also on their environmental sustainability and social impact-two key criteria highlighted in this paper. In doing so, this approach fosters an ethics of design in practice.

A crucial aspect to consider when assessing my proposal for an ethical morphospace is its evolutionary perspective on form (and on morphogenesis). Here, *form* is understood as the *construction and composition of multiple elements*, controlled by morphogenetic mechanisms, in line with the most recent morphological approach in evolutionary biology (Briggs, 2017; Tamborini, 2022b, 2021; Rieppel, 2020; Love, 2006, 2003). Forms are not optimal solutions but rather evolutionary compromises, as evolution is not an “optimizer but a satisfier”^11^. Consequently, the forms visualized and later produced through morphospace do not represent the most optimal ones, but rather a “general form”, as Raup observed (Raup, 1967): a working model that satisfies certain constraints.

One of the most delicate aspects of integrating an ethical dimension into morphospace is governance (Floridi, 2021, 2023; Coeckelbergh, 2024, 2006; Meisch et al., 2011; Tamborini, 2025b): who ultimately decides which forms are acceptable and which ethical parameters should be implemented into the morphospace? The answer to this question requires reflecting on how knowledge is produced in these disciplines, in effect supporting the agenda of philosophy and ethics of science in practice. As previously noted, in this kind of bio-inspired disciplines knowledge is shaped through hybridization: through knowledge circulation (Tamborini, 2024b, 2024b).

It is therefore essential to set up interdisciplinary ethics committees made up of scientists, philosophers, engineers and policymakers, and to introduce regular review mechanisms. These structures should continuously update ethical parameters in response to scientific and technological advancements and assess the acceptability of the proposed ethical criteria. In other words, ethical standards must evolve dynamically through the intersection of different perspectives and expertise.

Therefore, to operationalize and implement the proposed ethical morphospace, further interdisciplinary research is needed in at least three key areas:

1. Quantification of ethical parameters: How can we measure the ethical impact of different forms?
2. Normative Standards: What criteria should guide the selection of acceptable forms?
3. Social Participation: How can we integrate society’s voice in defining the ethical dimension of morphospace?

Hence and to conclude, in this paper, I have proposed a method for integrating ethical parameters into design. Architecture and bioengineering should not merely explore possible forms; they must also ask which forms are desirable for a sustainable future. By transforming morphospace from a purely feasibility-driven model into one that incorporates ethical responsibility, this work seeks to bridge the gap between technological possibility, feasibility, and moral necessity in design and engineering. Finally, this paper calls for new forms of interdisciplinary collaboration to discuss the ethical constraints that may operate in the morphospace.

## Data Availability

Not applicable.

## Footnotes

1 On the use and development morphospace and architectural design see (Steadman, 2008).

2 A terminological note. There is a strong relationship between *morphospace* and *design space*, as the latter is often used as a synonym for the former in the bio-design and computational design literature. The key distinction lies in their conceptual foundations: *morphospace* is form-centric and biologically grounded (see my arguments in this paper), whereas *design space* is problem–solution-oriented and performance-focused. In contemporary bio-design and computational architecture, however, these boundaries have become increasingly blurred as biological principles are progressively integrated into engineering design processes. For the purposes of this paper, I deliberately use only the term *morphospace*, as my proposal specifically builds on the biological and evolutionary foundations of form generation. Moreover, by using the notion of morphospace, I stress that the ethical parameters function as *morphogenetic constraints* rather than merely as design optimization criteria.

3 As I state in the outlook, this paper is therefore meant as a call for cooperation: it invites computational designers, engineers, philosophers, and policy-makers to co-develop the methods, metrics, and governance structures required to responsibly embed ethics into morphospace practice.

4 An ambulacrum is an elongated area of the shell of an echinoderm in which tubular feet are visible. The area between ambulacra is known as an interambulacrum.

5 Fabrication constraint is not the only one intervening in the architectural morphospace. See for instance (Mayencourt et al., 2025; Brown & Mueller, 2019; Brown et al., 2020; Łochnicki et al., 2022).

6 This is precisely the domain of *ex-ante* Life Cycle Assessment (LCA), a growing body of work that develops prospective models to estimate embodied carbon, energy use, and material circularity at early design stages. These methods address the challenge of allocating production and end-of-life impacts across multiple life cycles, which is particularly relevant for circular-economy strategies such as design-for-reuse. Integrating such models into morphospace generation allows environmental constraints to act as *ex ante* filters—excluding high-impact solutions before they are even generated—or as parameters that can be visualized and optimized alongside structural and economic criteria. See for instance (Cucurachi et al., 2018; Küpfer et al., 2021; Brütting et al., 2020; Wolf et al., 2020; Brütting et al., 2020)

7 While Value Sensitive Design (VSD) offers a powerful framework for systematically integrating ethical values into technology development, it has some limitations. For instance, one challenge is that VSD aspires to provide a “transcultural” platform for value integration, yet its own conceptual foundations are historically and culturally situated: it presupposes, for example, broadly liberal-democratic commitments such as individual autonomy, informed consent, and participatory deliberation. These commitments, while defensible, are not universally shared and may conflict with alternative ethical traditions. In the context of morphospace design, this means that the process of selecting which values to embed—and how to operationalize them—should explicitly involve negotiation across cultural, institutional, and disciplinary perspectives, rather than assuming a fixed universal set of values. See the outlook of this paper.

8 On Cassirer see (Cassirer, 2012; Hoel & der Tuin, 2013; Müller, 2010; Tamborini, 2025a)

9 This contribution is deliberately conceptual and normative rather than technical. Its aim is to propose a philosophical framework for integrating ethical considerations into morphospace practice and to justify why and where such integration is crucial. The result of this work is thus the creation of a conceptual foundation for the kind of quantitative implementations that future technical research can deliver. I would like to thank one referee for encouraging me to make this distinction.

10 On the possible technical implementation, insights can be drawn from Nagy Makhlouf’s work on visualizing financial constraints and from Balthazar Auguste-Dormeuil’s *The Cloud and the Sea* (2019), which “uses parametric coding to make visible and question mechanisms that cannot be physically experienced” (Makhlouf, 2025, p. 242).

11 I am borrowing this from Josh Bongard.
